# Report of an attack on a howler monkey *Alouatta sara* by a group of collared peccaries *Dicotyles tajacu* at a mammal clay lick in Madre de Dios, Peru

**DOI:** 10.5194/pb-9-29-2022

**Published:** 2022-09-01

**Authors:** Raul Bello, Eckhard Heymann, Sam Pottie

**Affiliations:** 1 Kawsay Biological Station, Madre de Dios, Peru; 2 German Primate Center, Göttingen, Germany; 3 Para La Tierra, Pilar, Paraguay

## Abstract

Howler monkeys *Alouatta* are almost exclusively arboreal. They will, however, occasionally descend to the forest floor to conduct geophagy at clay licks if these are present within their home range. They do this to incorporate certain minerals into their diet and/or for detoxification purposes. Clay licks are risky areas however, especially for arboreal mammals, as visiting them requires the monkeys to leave the safety of the trees. This has been confirmed by observed predation attempts on howler monkeys by large felines at clay licks. We report an additional risk for howler monkeys descending to the forest floor that has not previously been considered, namely potential attacks by collared peccaries *Dicotyles tajacu*. Camera traps were placed at three different clay licks in the Taricaya Ecological Reserve, located in the southeastern Peruvian Amazon, to monitor the fauna within the reserve. On 4 June 2017, the camera traps registered a lethal attack on a howler monkey by a group of collared peccaries at one of the clay licks.

## 1 Introduction

Clay licks are specific sites of interest within tropical ecosystems that are especially common in the western Amazon basin (Lee et al., 2009). They consist of exposed soil that contain relatively high percentages of certain minerals, such as sodium, calcium, potassium and iron (Brightsmith and Munoz-Najar, 2004; Emmons and Starck, 1979). Many animal species have been observed to intentionally consume these soils, a behaviour called geophagy (Klaus and Schmid, 1998). They do this for detoxification purposes and/or to supplement their diet with much-needed minerals, which are not easily available to them from other food sources (Johns and Duquette, 1991; Gilardi et al., 1999). These minerals are very difficult to obtain through a plant-based diet, which might explain why geophagy is highly biased towards frugivores and folivores (Kreulen, 1985). This results in high abundances of herbivores in and around clay licks (Klaus and Schmid, 1998), which in turn leads to increased risks of predation as predators are known to visit clay licks in search of prey (Matsuda and Izawa, 2008). Arboreal primates are especially vulnerable to predation at clay licks as such a visit requires them to descend to the forest floor (Janson, 1998). Attempted predation events by large felines on primates at clay licks have been reported (Matsuda and Izawa, 2008). Primate species have therefore evolved ways to mitigate this increased predation risk (Link et al., 2011). Howler monkeys, for example, have been observed to show prolonged vigilant behaviour before descending to ground level at clay licks (Izawa, 1993).

Apart from feline predators, other animals could also represent unexpected risks to clay-lick visitors. Here we report such an unexpected risk, namely the deadly attack of collared peccaries *Dicotyles tajacu* on a Bolivian red howler monkey *Alouatta sara* at a clay lick.

## 2 Methods

Bushnell Trophy Cam HD camera traps were used to monitor three different clay licks within the Taricaya Ecological Reserve (TER) as part of an ongoing fauna monitoring programme. TER is a 476 ha privately owned reserve located in the buffer zone of the Tambopata National Reserve, in Madre de Dios, Peru, and consists of seasonally flooded, primary, subtropical, wet forest according to the Holdridge life zone system (Holdridge, 1967). The monitored clay licks are forest-interior clay licks (Montenegro, 2004), and each one covers an area of between 40 and 70 m ${}^{2}$ . The camera traps were set to take videos of 10–20 s with a delay between videos of 10 s. No ethical consent was required for this study, as no animals had to be trapped or handled.

## 3 Results

On the 16 April 2017, the camera traps registered a group of collared peccaries *Dicotyles tajacu* and a group of howler monkeys *Alouatta sara*, using the same clay lick within close proximity of one another for a time period of approximately 41 min (08:41–09:22, all times given in local time). During this time, a minimum of eight peccaries and six howler monkeys were observed. At the start, only the peccaries were present in the clay lick. After a couple of minutes however, a large male howler monkey could be seen running on the ground, just outside the clay lick, and climbing up a tree. This male howler monkey climbed down again after some minutes and started to look for a spot between the peccaries inside the clay lick to feed on the clay. The howler monkey approached the peccaries to within 1 m while doing this, which resulted in three agonistic events carried out by the peccaries towards the howler monkey. These events consisted of sudden quick movements of the peccaries towards the howler monkey, combined with warning vocalizations. At 08:56, the male howler monkey eventually found a spot to feed on the clay in between the peccaries. Other howler monkeys started to come down the trees and could be seen feeding on the clay on the other side of the clay lick. The peccaries eventually started to leave, and when only two peccaries were left at around 09:09, the howler monkeys moved to the other side of the clay lick, where the two peccaries and the large male howler monkey were located. All animals positioned themselves at the right side of the large male howler monkey, away from the peccaries. At 09:16, only one peccary was left at the clay lick. This individual could be seen smelling the head of the large male howler monkey in one instance. At 09:22, the last peccary left the clay lick, and the howler monkeys appeared to be leaving shortly after.

On 4 June 2017 at 08:38, a subadult female howler monkey was attacked by a group of collared peccaries at the same clay lick. The video of the attack shows three peccaries and one howler monkey. The howler monkey appears hurt and is seen in a corner of the clay lick trying to escape. Before the howler monkey can exit the clay lick however, one of the peccaries can be seen attacking the howler monkey with its tusks. A different peccary then runs over the injured howler monkey, and finally the same peccary who attacked the howler monkey with its tusks at the start of the video can be seen to grab the howler monkey in its mouth and shake it violently, before throwing it on the ground. The howler monkey does not appear to move after landing on the ground, and this is where the video ends. The next videos are taken the following day between 12:21 and 12:25 and show two collared peccaries smelling, biting and pushing the carcass but not feeding on it.

Three days after the occurrence of the attack, when the camera traps were checked, the corpse of the howler monkey was collected and a necropsy was conducted to investigate the exact causes of death – we had not yet reviewed the camera-trap videos and were therefore not yet aware of the howler monkey attack. The necropsy found that the animal weighed 3.4 kg and suffered multiple internal and external traumas. A hypovolemic shock and traumatic respiratory deficiency were concluded to be the causes of death. No signs of carcass consumption were found during the necropsy.

## 4 Discussion

This attack confirms the sometimes unexpected risks primates face when descending to the forest floor for soil consumption. Neither the camera traps nor the necropsy registered any evidence of the peccaries feeding on the howler monkey carcass, even though collared peccaries' diet can consist of up to 18 % of animal matter in Neotropical forests (Aquino et al., 2001). Pedro Perez (personal communication, 1 September 2021) even reports them to be opportunistic carnivores of larger mammal species as he has observed collared peccaries feeding on the carcass of *Mazama americana* and *Cuniculus paca* in Loreto, Peru. Our results indicate that the observed attack was not a predation event but rather the result of the typical aggressive behaviour of peccaries (Mayor et al., 2006; Lochmiller and Grant, 1982). It is, however, important to note that the typical aggressive behaviour associated with peccaries is usually related to the white-lipped peccary *Tayassu pecari* and not with the collared peccary as various studies have found the latter to display more cautious behaviour (Nogueira et al., 2017).

It is not possible to identify the exact causes of this attack, but a possible explanation could be related to the subadult female not respecting a minimum critical distance between itself and the peccaries. This behaviour could have been a result of the subadult female howler monkey observing the large male howler monkey being in close contact with the peccaries for several minutes on the 16 April 2017. This might have given the subadult female a false sense of security around the peccaries. It is likely, however, that the difference in body size between the large male howler monkey and the subadult female may explain the very different reactions from the collared peccaries. This was most likely an uncommon event but is still a clear confirmation of the sometimes surprising risks geophagy can hold for primates.

## Data Availability

The camera-trap video of the attack was deposited in a reliable public data repository and can be accessed following the DOI in the “Video supplement” section.

## Video supplement

The camera-trap video of the attack is available under the following DOI: .

## References

[bib1.bib1] Aquino R, Bodmer R, Gil J (2001). Mamiferos de la cuenca del rio Samiria: Ecologia poblacional y sustentabilidad de la caza, Wildlife Conservation Society, Lima, Peru.

[bib1.bib2] Brightsmith D, Munoz-Najar R (2004). Avian geophagy and soil characteristics in southeastern Peru. Biotropica.

[bib1.bib3] Pottie S (2022). Peccary attack [video].

[bib1.bib4] Emmons L, Starck N (1979). Element composition of a natural mineral lick in Amazonia. Biotropica.

[bib1.bib5] Gilardi J, Duffey S, Jun C, Tell L (1999). Biochemical functions of geophagy in parrots: detoxification of dietary toxins and cryptoprotective effects. J Chem Ecol.

[bib1.bib6] Holdridge L (1967). Life zone ecology.

[bib1.bib7] Izawa K (1993). Soil-eating by *Alouatta* and *Ateles*. Int J Primatol.

[bib1.bib8] Janson C (1998). Testing the predation hypothesis for vertebrate sociality: prospects and pitfalls. Behaviour.

[bib1.bib9] Johns T, Duquette M (1991). Detoxification and mineral supplementations as functions of geophagy. Am J Clin Nutr.

[bib1.bib10] Klaus G, Schmid B (1998). Geophagy at natural licks and mammal ecology: a review. Mammalia.

[bib1.bib11] Kreulen D (1985). Lick use by large herbivores: a review of benefits and banes of soil consumption. Mammal Review.

[bib1.bib12] Lee A, Kumar S, Brightsmith D, Marsden S (2009). Parrot claylick distribution in South America: do patterns of “where” help answer the question of “why”?. Ecography.

[bib1.bib13] Link A, Galvis N, Fleming E, Di Fiore A (2011). Patterns of Mineral Lick Visitation by Spider Monkeys and Howler Monkeys in Amazonia: Are Licks Perceived as Risky Areas?. Am J Primatol.

[bib1.bib14] Lochmiller R, Grant W (1982). Intraspecific aggression results in death of a collared peccary. Zoo Biol.

[bib1.bib15] Matsuda I, Izawa K (2008). Predation of wild spider monkeys at La Macarena, Colombia. Primates.

[bib1.bib16] Mayor P, Le Pendu Y, Guimaraes D, da Silva J, Tavares H, Tello M, Pereira W, Lopez-Bejar M, Jori F (2006). A health evaluation in a colony of captive collared peccaries (*Tayassu tajacu*) in the eastern Amazon. R Vet Sci.

[bib1.bib17] Montenegro O (2004). >Natural licks as keystone resources for wildlife and people in Amazonia.

[bib1.bib18] Nogueira S, Reis A, Marsaro S, Duarte J, Moreto V, Lima S, Costa T, Nogueira-Filho S (2017). The defensive behavioral patterns of captive white-lipped and collared peccary (Mammalia,
*Tayassuidae*): an approach for conservation of the species. Acta Etholog.

